# Crystal structure of bis­[*N*,*N*-bis­(2-hydroxy­eth­yl)glycinato-κ^3^ *O* ^1^,*N*,*O* ^2^]cobalt(II) monohydrate

**DOI:** 10.1107/S205698901501943X

**Published:** 2015-10-17

**Authors:** Yang Liu, Dan Zhou, Hai-Hui Liu, Chen-Cong He

**Affiliations:** a Key Laboratory of Functional Organometallic Materials, Department of Chemistry and Materials Science, Hengyang Normal University, Hengyang 421008, People’s Republic of China; b Department of Chemistry and Materials Science, Hengyang Normal University, Hengyang 421008, People’s Republic of China

**Keywords:** crystal structure, *N*,*N*-bis­(2-hy­droxy­eth­yl)glycine, hydrogen bond

## Abstract

The title compound, [Co(C_6_H_12_O_4_)_2_]·H_2_O, was prepared by mild heating of an aqueous solution. The Co^II^ ion has a slightly distorted octahedral coordination environment which is defined by two N atoms occupying the apical position, while the equatorial plane is furnished by two hy­droxy O atoms and two carboxyl­ate O atoms. The four hy­droxy O atoms from two distinct *N*,*N*-bis­(2-hy­droxy­eth­yl)glycine (bicH_2_^−^) ligands act as hydrogen-bond donors with two carboxyl­ate O atoms as acceptors to form O—H⋯O hydrogen-bonded layers extending parallel to (100). In addition, the guest water mol­ecule acts as both a hydrogen-bond donor and acceptor, so that each Co(bicH_2_)_2_ mol­ecule is connected simultaneously to six neighbouring Co(bicH_2_)_2_ and two guest water mol­ecules by hydrogen bonding.

## Related literature   

For *N*,*N*-bis­(2-hy­droxy­eth­yl)glycine complexes with transition metals, see: Graham *et al.* (2009 ▸); Katsoulakou *et al.* (2011 ▸); Liu *et al.* (2013 ▸); Inomata *et al.* (2001 ▸); Messimeri *et al.* (2002 ▸). Iminodi­acetic acid (Cui *et al.*, 2008 ▸; Kong *et al.*, 2008 ▸), nitrilo­tri­acetic acid (Ma *et al.*, 2009 ▸) and *N*-(2-carbamoyl­meth­yl)iminodi­acetic acid (Bugella-Altamirano *et al.*, 2003 ▸) are also known to be effective ligands for transition metal ions. 
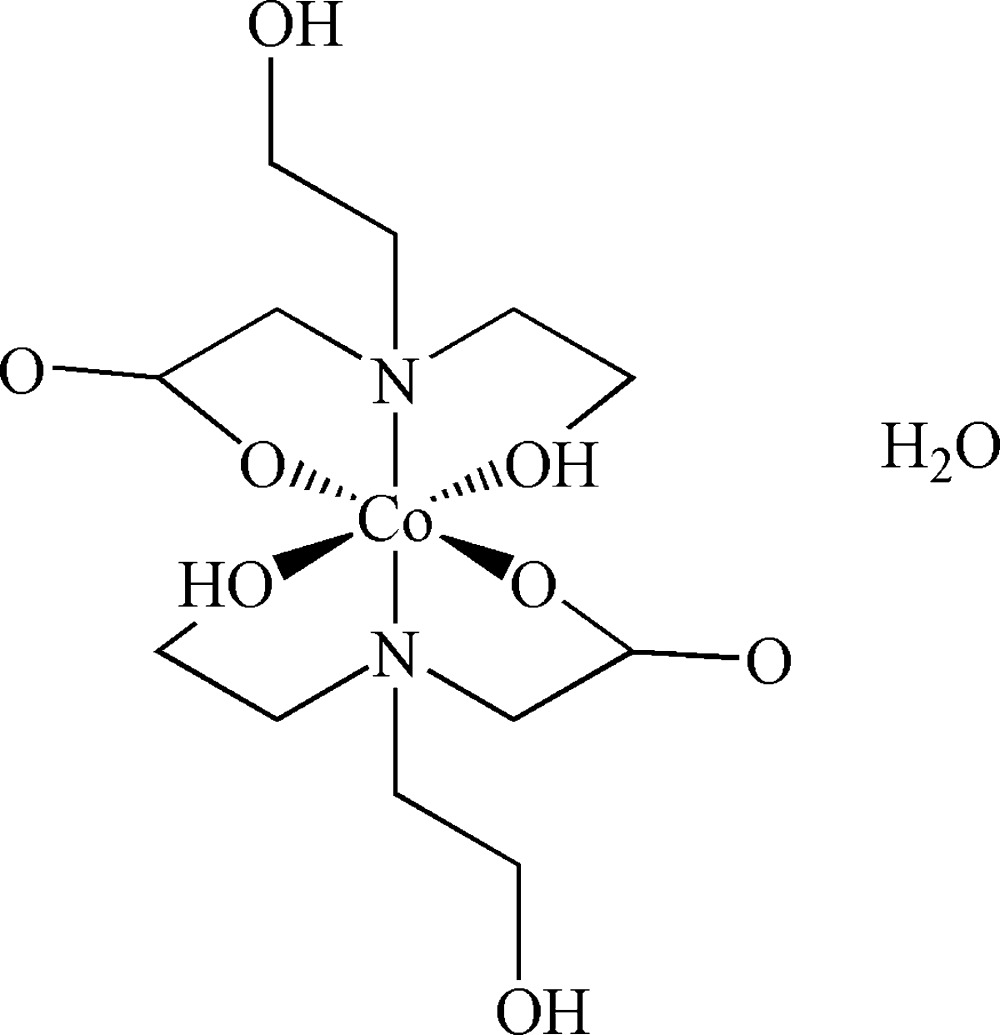


## Experimental   

### Crystal data  

[Co(C_6_H_12_NO_4_)_2_]·H_2_O
*M* *_r_* = 401.28
Monoclinic, 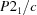

*a* = 19.274 (3) Å
*b* = 12.0033 (17) Å
*c* = 7.196 (1) Å
β = 100.081 (2)°
*V* = 1639.1 (4) Å^3^
*Z* = 4
Mo *K*α radiation
μ = 1.10 mm^−1^
*T* = 296 K
0.20 × 0.20 × 0.20 mm

### Data collection  

Bruker SMART CCD area-detector diffractometer
Absorption correction: multi-scan (*SADABS*; Bruker, 2012 ▸) *T*_min_ = 0.810, *T*_max_ = 0.810
9415 measured reflections
3685 independent reflections
2982 reflections with *I* > 2σ(*I*)
*R*_int_ = 0.031

### Refinement  

*R*[*F* ^2^ > 2σ(*F* ^2^)] = 0.037
*wR*(*F* ^2^) = 0.108
*S* = 1.04
3685 reflections
229 parameters
7 restraints
H atoms treated by a mixture of independent and constrained refinement
Δρ_max_ = 1.22 e Å^−3^
Δρ_min_ = −0.51 e Å^−3^

### 

Data collection: *SMART* (Bruker, 2012 ▸); cell refinement: *SAINT* (Bruker, 2012 ▸); data reduction: *SAINT*; program(s) used to solve structure: *SHELXS97* (Sheldrick, 2008 ▸); program(s) used to refine structure: *SHELXL97* (Sheldrick, 2008 ▸); molecular graphics: *ORTEP-3 for Windows* (Farrugia, 2012 ▸); software used to prepare material for publication: *SHELXTL* (Sheldrick, 2008 ▸).

## Supplementary Material

Crystal structure: contains datablock(s) I, New_Global_Publ_Block. DOI: 10.1107/S205698901501943X/bq2401sup1.cif

Structure factors: contains datablock(s) I. DOI: 10.1107/S205698901501943X/bq2401Isup2.hkl

Supporting information file. DOI: 10.1107/S205698901501943X/bq2401Isup3.docx

**Fig. 1.**
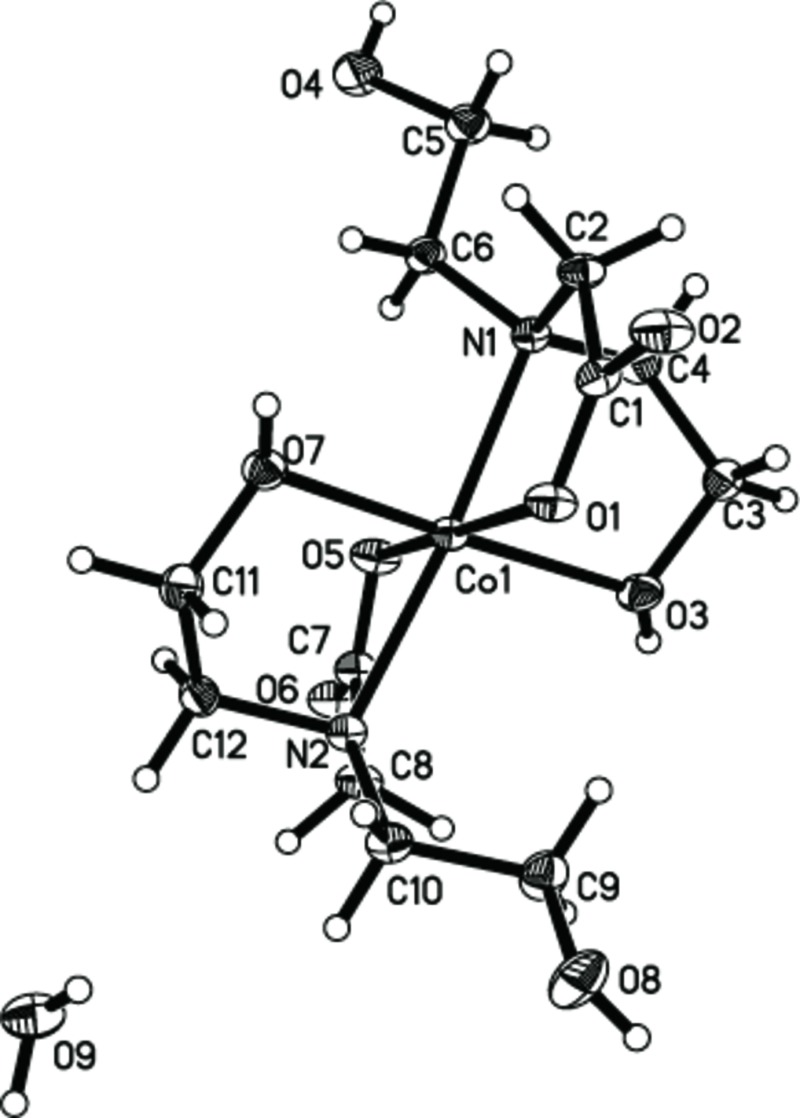
The structure of the title complex, showing 30% probability displacement ellipsoids and the atom-numbering scheme. H atoms have been omitted for clarity.

**Fig. 2.**
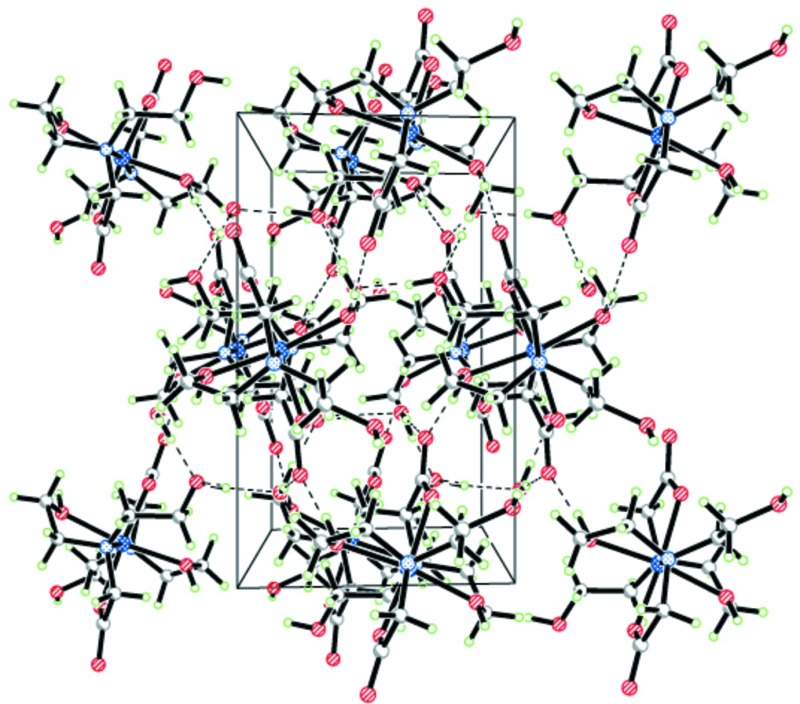
A partial view along the c axis of the crystal packing of the title compound.

. DOI: 10.1107/S205698901501943X/bq2401fig1.tif
The structure of the title complex, showing 30% probability displacement ellipsoids and the atom-numbering scheme. H atoms have been omitted for clarity.

c . DOI: 10.1107/S205698901501943X/bq2401fig2.tif
A partial view along the *c* axis of the crystal packing of the title compound.

**Fig. 3.**
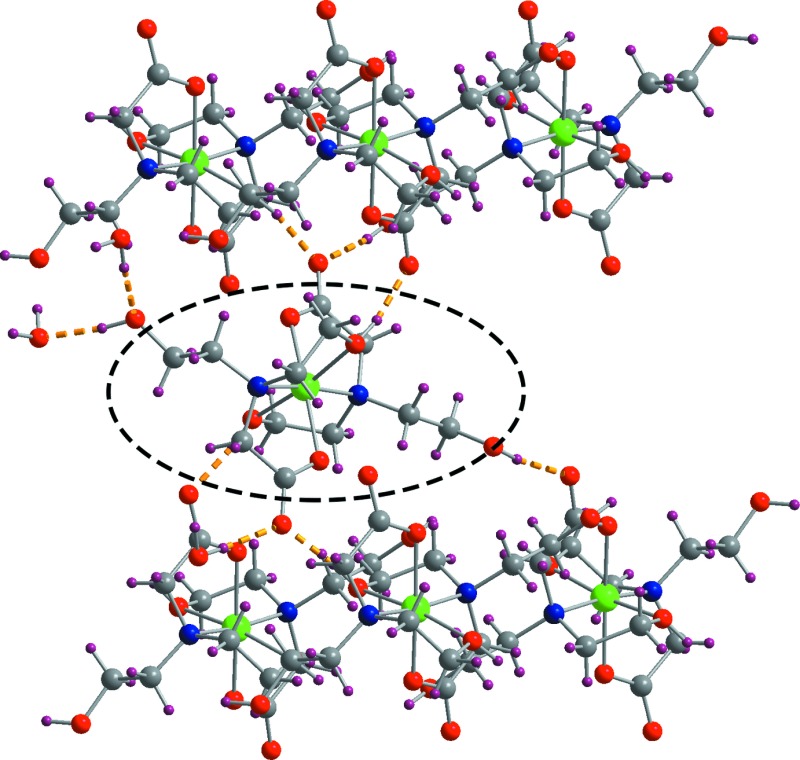
View of the hydrogen-bonding interactions for the title compound.

. DOI: 10.1107/S205698901501943X/bq2401fig3.tif
View of the hydrogen-bonding inter­actions for the title compound.

CCDC reference: 1431271

Additional supporting information:  crystallographic information; 3D view; checkCIF report

## Figures and Tables

**Table 1:** Hydrogen-bond geometry (, )

*D*H*A*	*D*H	H*A*	*D* *A*	*D*H*A*
O3H3*AA*O6^i^	0.81(2)	1.79(2)	2.591(2)	169(3)
O4H4*AA*O2^ii^	0.76(2)	1.98(2)	2.733(2)	171(3)
O7H7*AA*O2^iii^	0.82(2)	1.83(2)	2.648(2)	178(3)
O8H8*AA*O9^iv^	0.82(2)	1.89(2)	2.687(3)	162(3)
O9H9*AA*O6^v^	0.82(2)	1.99(2)	2.796(2)	171(3)
O9H9*BB*O8^iii^	0.81(2)	1.95(2)	2.759(3)	176(3)

Symmetry codes: (i) 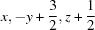; (ii) 
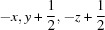
; (iii) 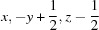; (iv) 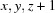; (v) 
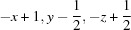
.

## supplementary crystallographic information

### S1. Comment

The design and synthesis of transition metal coordination complexes based on those multi-dentate flexible carboxyl­ate ligands have attracted significant attention due to their structural diversity and utility in supra­molecular chemistry and crystal engineering. Iminodi­acetic acid (Cui *et al.*, 2008; Kong *et al.*, 2008), nitrilo­tri­acetic acid (Ma *et al.*, 2009) and N-(2-carbamoyl­methyl)­iminodi­acetic acid (Bugella-Altamirano *et al.*, 2003) have been known as effective ligands for transition metal ions. As an analogous ligand, N,N-bis­(2-hy­droxy­ethyl) glycine is a widely used buffer in many biochemical studies. However, transition metal complexes with N,N-bis­(2-hy­droxy­ethyl) glycine has been less extensively studied, and only a few reports describing N,N-bis­(2-hy­droxy­ethyl) glycine complexes have appeared (Graham *et al.*, 2009; Katsoulakou *et al.*, 2011; Liu *et al*., 2013; Inomata *et al.*, 2001; Messimeri *et al.*, 2002). In the present report, we describe the synthesis and structure of title compound.

Single-crystal X-ray diffraction analysis shows that the title compound crystallizes in the monoclinic space group *P2_1_/c* and its asymmetric unit contains one Co (II) ion, two distinct deprotonated N,N-bis­(2-hy­droxy­ethyl) glycine (bicH_2_^-^) anions and one water molecule. As showed in Fig. 1, Co^II^ ion has a six-coordinated o­cta­hedral geometry which is defined by two nitro­gen atoms occupying the apical position, while the equatorial plane are furnished by two hydroxyl oxygen atoms and two carboxyl­ate atoms. The Co—O (Co1—O1 = 2.0544 (14) Å; Co1—O3 = 2.1093 Å; Co1—O5 = 2.0853 (15) Å; Co1—O7 = 2.1006 (7) Å) and Co—N (Co1—N1 = 2.1641 Å; Co1—N2 = 2.1881 Å) bond lengths are fall in the usual range. The crystal structure of the title compound is stabilized by hydrogen bonds. A packing diagram of the complex showing hydrogen bonding inter­actions is shown in Fig. 2. The hydroxyl oxygen atoms (O3, O4, O7, O8) from the N,N-bis­(2-hy­droxy­ethyl)­glycine ligands act as donors, while the carboxyl­ate oxygen atoms (O2 and O6) are the acceptors. The hydrogen bonding inter­actions around one molecule are shown in Fig. 3. The hydrogen bonding parameters are tabulated in Table 1.

### S2. Experimental

A mixture of CoCl_2_·6H_2_O (0.237g, 1mmol) and N,N-bis­(2-hy­droxy­ethyl) glycine; (0.16g, 1mmol) was dissolved in water (20mL) and then drop of ethlylene di­amine was added, and the mixture was stirred vigorously for 1h at 60 °C. Slow evaporation of the clear solution resulted in the separation of blue block crystals.

### S3. Refinement

All H atoms were positioned geometrically and treated as riding on their parent atoms [C—H =0.97 Å and *U*iso = 1.2Ueq (C) for CH_2_ H atoms].

### Crystal data

**Table:**

[Co(C_6_H_12_NO_4_)_2_]·H_2_O	*F*(000) = 844
*M**_r_* = 401.28	*D*_x_ = 1.626 Mg m^−^^3^
Monoclinic, *P*2_1_/*c*	Mo *K*α radiation, λ = 0.71073 Å
*a* = 19.274 (3) Å	Cell parameters from 4351 reflections
*b* = 12.0033 (17) Å	θ = 2.7–27.4°
*c* = 7.196 (1) Å	µ = 1.10 mm^−^^1^
β = 100.081 (2)°	*T* = 296 K
*V* = 1639.1 (4) Å^3^	Block, red
*Z* = 4	0.20 × 0.20 × 0.20 mm

### Data collection

**Table:**

Bruker SMART CCD area-detector diffractometer	3685 independent reflections
Radiation source: fine-focus sealed tube	2982 reflections with *I* > 2σ(*I*)
Graphite monochromator	*R*_int_ = 0.031
phi and ω scans	θ_max_ = 27.5°, θ_min_ = 1.1°
Absorption correction: multi-scan (*SADABS*; Bruker, 2012)	*h* = −25→24
*T*_min_ = 0.810, *T*_max_ = 0.810	*k* = −14→15
9415 measured reflections	*l* = −7→9

### Refinement

**Table:**

Refinement on *F*^2^	Primary atom site location: structure-invariant direct methods
Least-squares matrix: full	Secondary atom site location: difference Fourier map
*R*[*F*^2^ > 2σ(*F*^2^)] = 0.037	Hydrogen site location: inferred from neighbouring sites
*wR*(*F*^2^) = 0.108	H atoms treated by a mixture of independent and constrained refinement
*S* = 1.04	*w* = 1/[σ^2^(*F*_o_^2^) + (0.0683*P*)^2^ + 0.2267*P*] where *P* = (*F*_o_^2^ + 2*F*_c_^2^)/3
3685 reflections	(Δ/σ)_max_ < 0.001
229 parameters	Δρ_max_ = 1.22 e Å^−^^3^
7 restraints	Δρ_min_ = −0.51 e Å^−^^3^

### Special details

**Table:**

Geometry. All e.s.d.'s (except the e.s.d. in the dihedral angle between two l.s. planes) are estimated using the full covariance matrix. The cell e.s.d.'s are taken into account individually in the estimation of e.s.d.'s in distances, angles and torsion angles; correlations between e.s.d.'s in cell parameters are only used when they are defined by crystal symmetry. An approximate (isotropic) treatment of cell e.s.d.'s is used for estimating e.s.d.'s involving l.s. planes.
Refinement. Refinement of *F*^2^ against ALL reflections. The weighted *R*-factor *wR* and goodness of fit *S* are based on *F*^2^, conventional *R*-factors *R* are based on *F*, with *F* set to zero for negative *F*^2^. The threshold expression of *F*^2^ > σ(*F*^2^) is used only for calculating *R*-factors(gt) *etc*. and is not relevant to the choice of reflections for refinement. *R*-factors based on *F*^2^ are statistically about twice as large as those based on *F*, and *R*- factors based on ALL data will be even larger.

### Fractional atomic coordinates and isotropic or equivalent isotropic displacement parameters (Å^2^)

**Table:**

	*x*	*y*	*z*	*U*_iso_*/*U*_eq_	
C1	0.15613 (10)	0.32888 (16)	0.4861 (3)	0.0250 (4)	
C2	0.10124 (10)	0.41726 (16)	0.4166 (3)	0.0257 (4)	
H2A	0.0728	0.3921	0.2995	0.031*	
H2B	0.0703	0.4255	0.5086	0.031*	
C3	0.18533 (11)	0.56936 (19)	0.7134 (3)	0.0317 (5)	
H3A	0.1877	0.6247	0.8125	0.038*	
H3B	0.1715	0.4989	0.7620	0.038*	
C4	0.13146 (11)	0.60422 (18)	0.5450 (3)	0.0292 (5)	
H4A	0.0850	0.6048	0.5794	0.035*	
H4B	0.1419	0.6792	0.5075	0.035*	
C5	0.02221 (11)	0.61413 (19)	0.1906 (3)	0.0323 (5)	
H5A	−0.0073	0.5502	0.2042	0.039*	
H5B	0.0177	0.6676	0.2889	0.039*	
C6	0.09822 (6)	0.57870 (9)	0.20355 (17)	0.0258 (4)	
H6A	0.1008	0.5260	0.1029	0.031*	
H6B	0.1256	0.6436	0.1812	0.031*	
C7	0.32255 (6)	0.69375 (9)	0.32133 (17)	0.0248 (4)	
C8	0.37980 (6)	0.60795 (9)	0.38576 (17)	0.0260 (4)	
H8A	0.4193	0.6211	0.3217	0.031*	
H8B	0.3964	0.6158	0.5204	0.031*	
C9	0.40241 (13)	0.41472 (19)	0.6612 (3)	0.0364 (5)	
H9A	0.4220	0.4849	0.7126	0.044*	
H9B	0.3550	0.4077	0.6882	0.044*	
C10	0.40096 (11)	0.41103 (16)	0.4523 (3)	0.0264 (4)	
H10A	0.4483	0.4236	0.4281	0.032*	
H10B	0.3865	0.3371	0.4064	0.032*	
C11	0.29542 (11)	0.38393 (18)	0.0716 (3)	0.0304 (5)	
H11A	0.2917	0.3727	−0.0633	0.036*	
H11B	0.3093	0.3141	0.1353	0.036*	
C12	0.34855 (5)	0.47358 (8)	0.13899 (15)	0.0273 (4)	
H12A	0.3944	0.4512	0.1144	0.033*	
H12B	0.3352	0.5418	0.0698	0.033*	
N1	0.13172 (5)	0.52733 (8)	0.38473 (15)	0.0212 (3)	
N2	0.35292 (5)	0.49430 (8)	0.34461 (15)	0.0211 (4)	
O1	0.21990 (7)	0.34960 (12)	0.4875 (2)	0.0277 (3)	
O2	0.13358 (8)	0.23761 (12)	0.5359 (2)	0.0371 (4)	
O3	0.25265 (7)	0.55828 (13)	0.6590 (2)	0.0301 (3)	
O4	0.00231 (8)	0.66298 (18)	0.0101 (2)	0.0420 (5)	
O5	0.25988 (7)	0.66233 (12)	0.2898 (2)	0.0300 (3)	
O6	0.34269 (8)	0.79245 (11)	0.3073 (2)	0.0345 (4)	
O7	0.22955 (8)	0.42119 (12)	0.1150 (2)	0.0287 (3)	
O8	0.44467 (10)	0.32506 (15)	0.7424 (3)	0.0462 (5)	
O9	0.51436 (10)	0.34647 (15)	0.0981 (3)	0.0430 (4)	
Co1	0.242384 (13)	0.500933 (18)	0.37854 (4)	0.01991 (11)	
H3AA	0.2766 (14)	0.6113 (19)	0.700 (4)	0.051 (9)*	
H4AA	−0.0348 (10)	0.685 (2)	0.010 (4)	0.047 (8)*	
H7AA	0.1993 (14)	0.373 (2)	0.091 (5)	0.067 (10)*	
H8AA	0.4681 (15)	0.345 (3)	0.843 (3)	0.056 (9)*	
H9AA	0.5568 (9)	0.336 (2)	0.118 (4)	0.049 (8)*	
H9BB	0.4938 (13)	0.298 (2)	0.145 (4)	0.064 (11)*	

### Atomic displacement parameters (Å^2^)

**Table:**

	*U*^11^	*U*^22^	*U*^33^	*U*^12^	*U*^13^	*U*^23^
C1	0.0250 (10)	0.0219 (10)	0.0260 (10)	−0.0018 (8)	−0.0013 (8)	0.0019 (8)
C2	0.0209 (9)	0.0212 (10)	0.0333 (11)	−0.0024 (7)	0.0001 (8)	0.0042 (8)
C3	0.0303 (11)	0.0378 (12)	0.0264 (11)	−0.0023 (9)	0.0031 (8)	−0.0051 (9)
C4	0.0304 (11)	0.0256 (10)	0.0310 (11)	0.0031 (8)	0.0035 (9)	−0.0044 (9)
C5	0.0271 (11)	0.0337 (12)	0.0352 (12)	0.0057 (9)	0.0023 (9)	0.0055 (9)
C6	0.0234 (10)	0.0268 (10)	0.0262 (10)	0.0030 (8)	0.0016 (8)	0.0048 (8)
C7	0.0250 (10)	0.0187 (9)	0.0299 (10)	0.0005 (7)	0.0027 (8)	0.0034 (8)
C8	0.0215 (9)	0.0172 (9)	0.0380 (12)	−0.0020 (7)	0.0020 (8)	0.0013 (8)
C9	0.0405 (13)	0.0335 (12)	0.0339 (12)	0.0120 (10)	0.0029 (10)	0.0063 (9)
C10	0.0261 (10)	0.0194 (9)	0.0328 (11)	0.0056 (8)	0.0024 (8)	0.0013 (8)
C11	0.0316 (11)	0.0303 (11)	0.0291 (11)	0.0000 (9)	0.0049 (9)	−0.0059 (9)
C12	0.0259 (10)	0.0297 (10)	0.0270 (10)	0.0009 (8)	0.0069 (8)	0.0025 (8)
N1	0.0209 (8)	0.0174 (7)	0.0246 (8)	0.0018 (6)	0.0019 (6)	0.0010 (6)
N2	0.0216 (9)	0.0155 (8)	0.0254 (9)	0.0008 (6)	0.0025 (7)	0.0007 (6)
O1	0.0212 (7)	0.0206 (7)	0.0403 (8)	−0.0010 (5)	0.0021 (6)	0.0058 (6)
O2	0.0274 (8)	0.0254 (8)	0.0553 (10)	−0.0057 (6)	−0.0015 (7)	0.0155 (7)
O3	0.0254 (8)	0.0305 (9)	0.0324 (8)	−0.0043 (6)	−0.0006 (6)	−0.0082 (6)
O4	0.0300 (9)	0.0542 (12)	0.0399 (10)	0.0163 (8)	0.0011 (8)	0.0155 (7)
O5	0.0218 (7)	0.0213 (7)	0.0459 (9)	0.0004 (5)	0.0030 (6)	0.0069 (6)
O6	0.0283 (8)	0.0177 (7)	0.0546 (10)	−0.0021 (6)	−0.0012 (7)	0.0092 (6)
O7	0.0258 (8)	0.0288 (8)	0.0310 (8)	−0.0031 (6)	0.0035 (6)	−0.0057 (6)
O8	0.0596 (12)	0.0354 (9)	0.0381 (10)	0.0159 (8)	−0.0070 (8)	0.0072 (8)
O9	0.0337 (10)	0.0388 (10)	0.0527 (11)	0.0051 (8)	−0.0032 (8)	0.0065 (9)
Co1	0.01819 (17)	0.01581 (17)	0.02525 (18)	−0.00122 (8)	0.00248 (11)	0.00056 (9)

### Geometric parameters (Å, º)

**Table:**

C1—O1	1.252 (2)	C9—O8	1.413 (3)
C1—O2	1.254 (2)	C9—C10	1.499 (3)
C1—C2	1.520 (3)	C9—H9A	0.9700
C2—N1	1.480 (2)	C9—H9B	0.9700
C2—H2A	0.9700	C10—N2	1.485 (2)
C2—H2B	0.9700	C10—H10A	0.9700
C3—O3	1.426 (3)	C10—H10B	0.9700
C3—C4	1.510 (3)	C11—O7	1.431 (3)
C3—H3A	0.9700	C11—C12	1.506 (2)
C3—H3B	0.9700	C11—H11A	0.9700
C4—N1	1.478 (2)	C11—H11B	0.9700
C4—H4A	0.9700	C12—N2	1.4882
C4—H4B	0.9700	C12—H12A	0.9700
C5—O4	1.416 (3)	C12—H12B	0.9700
C5—C6	1.513 (2)	N1—Co1	2.1644
C5—H5A	0.9700	N2—Co1	2.1878
C5—H5B	0.9700	O1—Co1	2.0544 (14)
C6—N1	1.4840 (15)	O3—Co1	2.1083 (15)
C6—H6A	0.9700	O3—H3AA	0.811 (17)
C6—H6B	0.9700	O4—H4AA	0.761 (17)
C7—O5	1.2476 (17)	O5—Co1	2.0858 (14)
C7—O6	1.2562 (17)	O7—Co1	2.1000 (15)
C7—C8	1.5212	O7—H7AA	0.820 (18)
C8—N2	1.4709 (15)	O8—H8AA	0.822 (17)
C8—H8A	0.9700	O9—H9AA	0.815 (16)
C8—H8B	0.9700	O9—H9BB	0.810 (16)
			
O1—C1—O2	124.06 (18)	C9—C10—H10B	108.8
O1—C1—C2	119.33 (17)	H10A—C10—H10B	107.7
O2—C1—C2	116.60 (17)	O7—C11—C12	106.59 (15)
N1—C2—C1	113.69 (15)	O7—C11—H11A	110.4
N1—C2—H2A	108.8	C12—C11—H11A	110.4
C1—C2—H2A	108.8	O7—C11—H11B	110.4
N1—C2—H2B	108.8	C12—C11—H11B	110.4
C1—C2—H2B	108.8	H11A—C11—H11B	108.6
H2A—C2—H2B	107.7	N2—C12—C11	110.89 (9)
O3—C3—C4	109.65 (17)	N2—C12—H12A	109.5
O3—C3—H3A	109.7	C11—C12—H12A	109.5
C4—C3—H3A	109.7	N2—C12—H12B	109.5
O3—C3—H3B	109.7	C11—C12—H12B	109.5
C4—C3—H3B	109.7	H12A—C12—H12B	108.0
H3A—C3—H3B	108.2	C4—N1—C2	112.38 (14)
N1—C4—C3	110.93 (16)	C4—N1—C6	111.45 (11)
N1—C4—H4A	109.5	C2—N1—C6	112.57 (11)
C3—C4—H4A	109.5	C4—N1—Co1	104.21 (10)
N1—C4—H4B	109.5	C2—N1—Co1	106.97 (9)
C3—C4—H4B	109.5	C6—N1—Co1	108.76 (7)
H4A—C4—H4B	108.0	C8—N2—C10	110.71 (11)
O4—C5—C6	106.08 (16)	C8—N2—C12	108.16 (6)
O4—C5—H5A	110.5	C10—N2—C12	109.15 (9)
C6—C5—H5A	110.5	C8—N2—Co1	105.03 (7)
O4—C5—H5B	110.5	C10—N2—Co1	119.78 (10)
C6—C5—H5B	110.5	C12—N2—Co1	103.31 (3)
H5A—C5—H5B	108.7	C1—O1—Co1	116.41 (12)
N1—C6—C5	116.02 (12)	C3—O3—Co1	110.85 (11)
N1—C6—H6A	108.3	C3—O3—H3AA	108 (2)
C5—C6—H6A	108.3	Co1—O3—H3AA	124 (2)
N1—C6—H6B	108.3	C5—O4—H4AA	104 (2)
C5—C6—H6B	108.3	C7—O5—Co1	115.36 (10)
H6A—C6—H6B	107.4	C11—O7—Co1	111.77 (12)
O5—C7—O6	124.99 (13)	C11—O7—H7AA	111 (2)
O5—C7—C8	118.57 (8)	Co1—O7—H7AA	119 (2)
O6—C7—C8	116.43 (8)	C9—O8—H8AA	109 (2)
N2—C8—C7	110.82 (6)	H9AA—O9—H9BB	111 (2)
N2—C8—H8A	109.5	O1—Co1—O5	173.89 (6)
C7—C8—H8A	109.5	O1—Co1—O7	86.68 (6)
N2—C8—H8B	109.5	O5—Co1—O7	98.43 (6)
C7—C8—H8B	109.5	O1—Co1—O3	85.08 (6)
H8A—C8—H8B	108.1	O5—Co1—O3	89.81 (6)
O8—C9—C10	107.49 (18)	O7—Co1—O3	171.76 (6)
O8—C9—H9A	110.2	O1—Co1—N1	81.21 (5)
C10—C9—H9A	110.2	O5—Co1—N1	94.77 (5)
O8—C9—H9B	110.2	O7—Co1—N1	97.16 (5)
C10—C9—H9B	110.2	O3—Co1—N1	81.97 (5)
H9A—C9—H9B	108.5	O1—Co1—N2	106.55 (5)
N2—C10—C9	113.81 (16)	O5—Co1—N2	77.69 (5)
N2—C10—H10A	108.8	O7—Co1—N2	81.12 (5)
C9—C10—H10A	108.8	O3—Co1—N2	100.87 (5)
N2—C10—H10B	108.8	N1—Co1—N2	171.86 (5)

### Hydrogen-bond geometry (Å, º)

**Table:**

*D*—H···*A*	*D*—H	H···*A*	*D*···*A*	*D*—H···*A*
O3—H3*AA*···O6^i^	0.81 (2)	1.79 (2)	2.591 (2)	169 (3)
O4—H4*AA*···O2^ii^	0.76 (2)	1.98 (2)	2.733 (2)	171 (3)
O7—H7*AA*···O2^iii^	0.82 (2)	1.83 (2)	2.648 (2)	178 (3)
O8—H8*AA*···O9^iv^	0.82 (2)	1.89 (2)	2.687 (3)	162 (3)
O9—H9*AA*···O6^v^	0.82 (2)	1.99 (2)	2.796 (2)	171 (3)
O9—H9*BB*···O8^iii^	0.81 (2)	1.95 (2)	2.759 (3)	176 (3)

Symmetry codes: (i) *x*, −*y*+3/2, *z*+1/2; (ii) −*x*, *y*+1/2, −*z*+1/2; (iii) *x*, −*y*+1/2, *z*−1/2; (iv) *x*, *y*, *z*+1; (v) −*x*+1, *y*−1/2, −*z*+1/2.
