# Supplementary material for: Crystal structure of bis­[N,N-bis­(2-hydroxy­eth­yl)glycinato-κ3 O 1,N,O 2]cobalt(II) monohydrate
Source: Acta Crystallogr E Crystallogr Commun. 2015 Oct 17;71(Pt 11):m199–200. doi: 10.1107/S205698901501943X (PMC4645093; doi:10.1107/S205698901501943X)
Supplement: Supplementary file 3 [file e-71-0m199-Isup3.docx]

Table 1

Hydrogen-bond geometry (Å, °)

| D-H···A | d(D-H) | d(H···A) | d(D···A) | <(DHA) |
| --- | --- | --- | --- | --- |
| O3-H3AA···O6^i^ | 0.811 | 1.791 | 2.591 | 168.50 |
| O4-H4AA···O2^ii^ | 0.761 | 1.979 | 2.733 | 170.64 |
| O7-H7AA···O2^iii^ | 0.820 | 1.828 | 2.648 | 178.44 |
| O8-H8AA···O9^iv^ | 0.822 | 1.894 | 2.687 | 161.81 |
| O9-H9AA···O6^v^ | 0.815 | 1.988 | 2.796 | 171.29 |
| O9-H9BB···O8^iii^ | 0.810 | 1.950 | 2.759 | 176.34 |

Symmetry codes:

(i)x,-y+3/2,z+1/2;(ii)-x,y+1/2,-z+1/2; (iii)x,-y+1/2,z-1/2; (iv) x, y, z+1; (v) -x+1, y-1/2, -z+1/2
