# Supplementary figures and images for: Crystal structure of bis­[N,N-bis­(2-hydroxy­eth­yl)glycinato-κ3 O 1,N,O 2]cobalt(II) monohydrate
Source: Acta Crystallogr E Crystallogr Commun. 2015 Oct 17;71(Pt 11):m199–200. doi: 10.1107/S205698901501943X (PMC4645093; doi:10.1107/S205698901501943X)

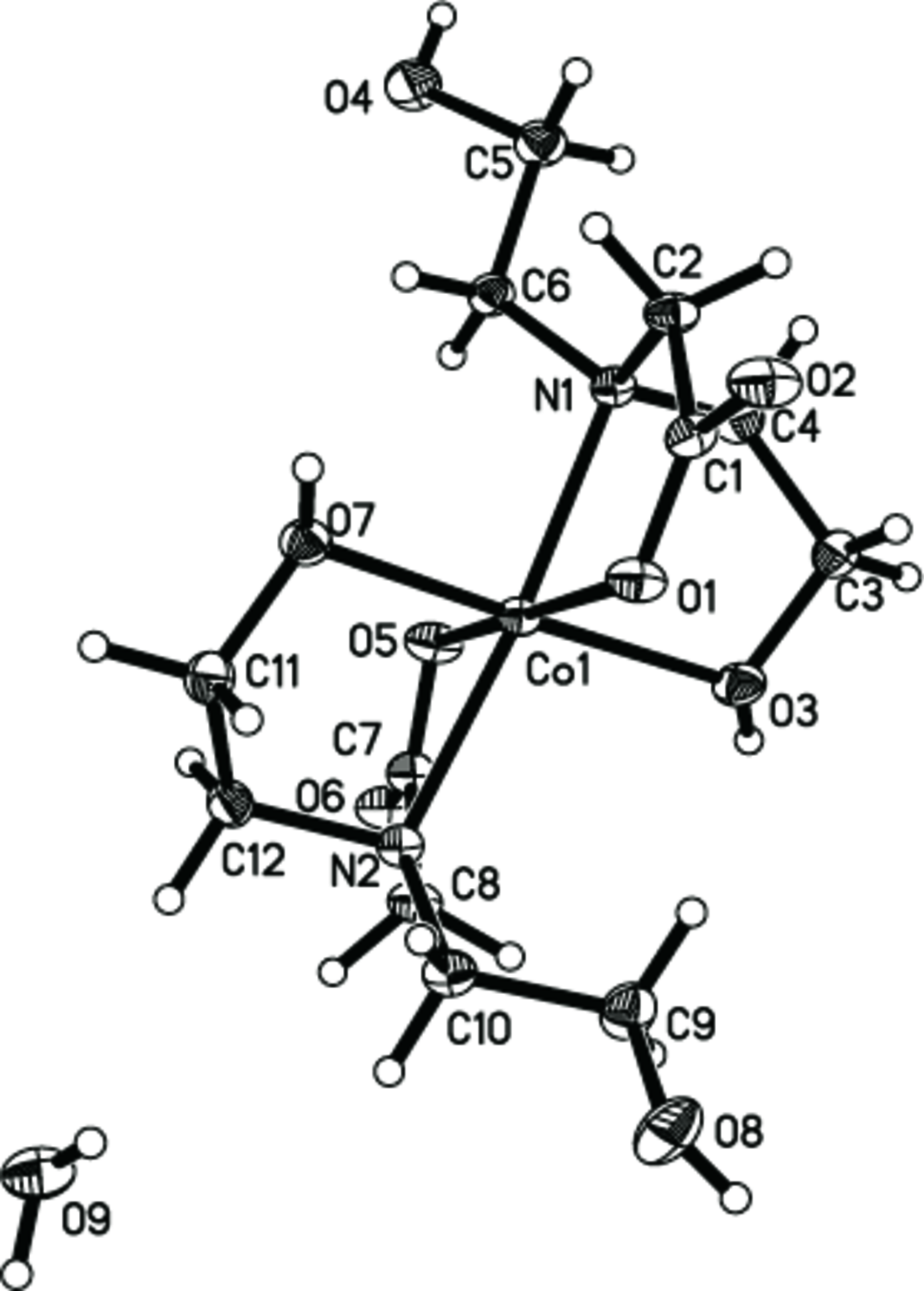

Supplement: Supplementary file 4 [file e-71-0m199-fig1.tif]

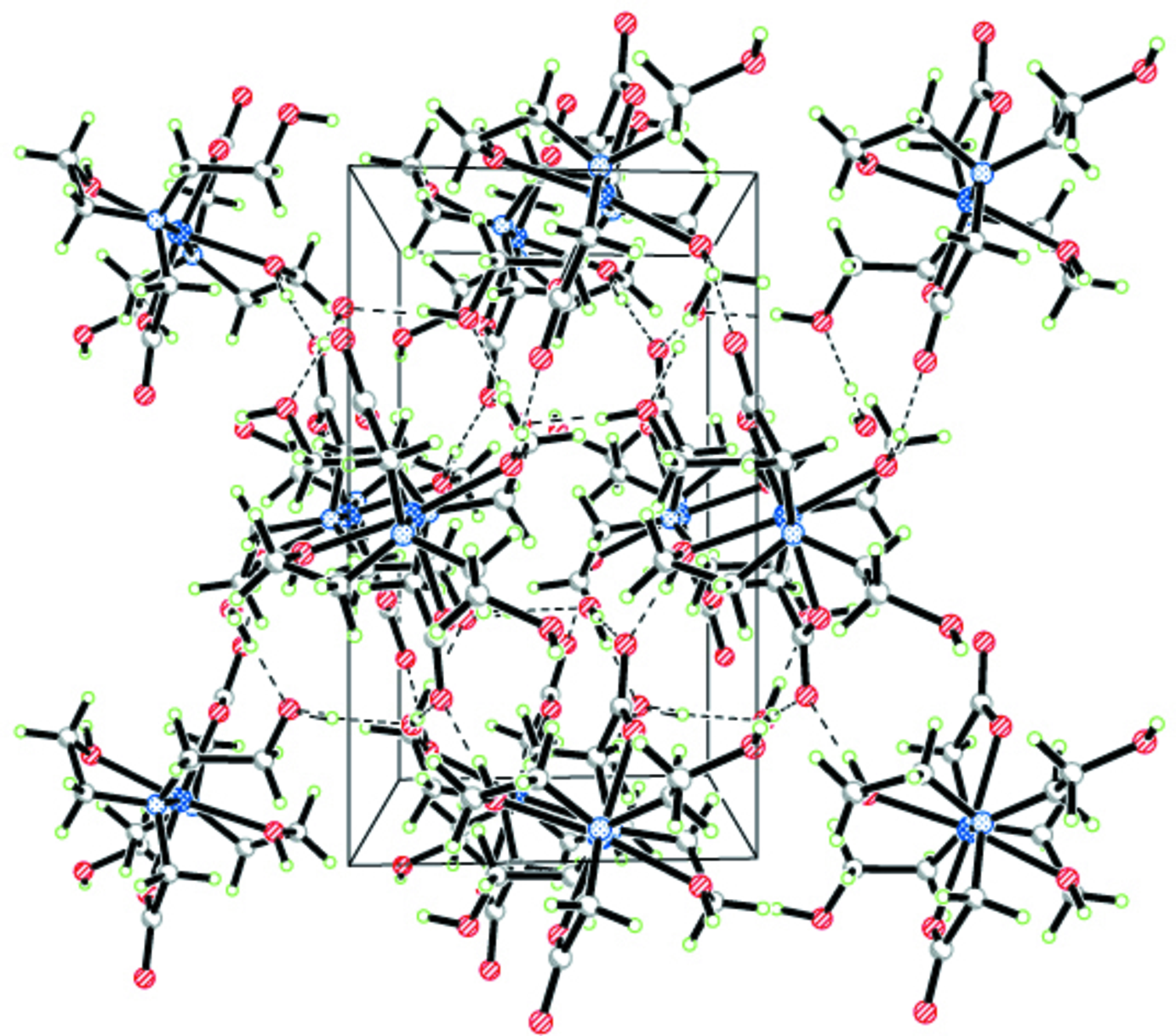

Supplement: Supplementary file 5 [file e-71-0m199-fig2.tif]

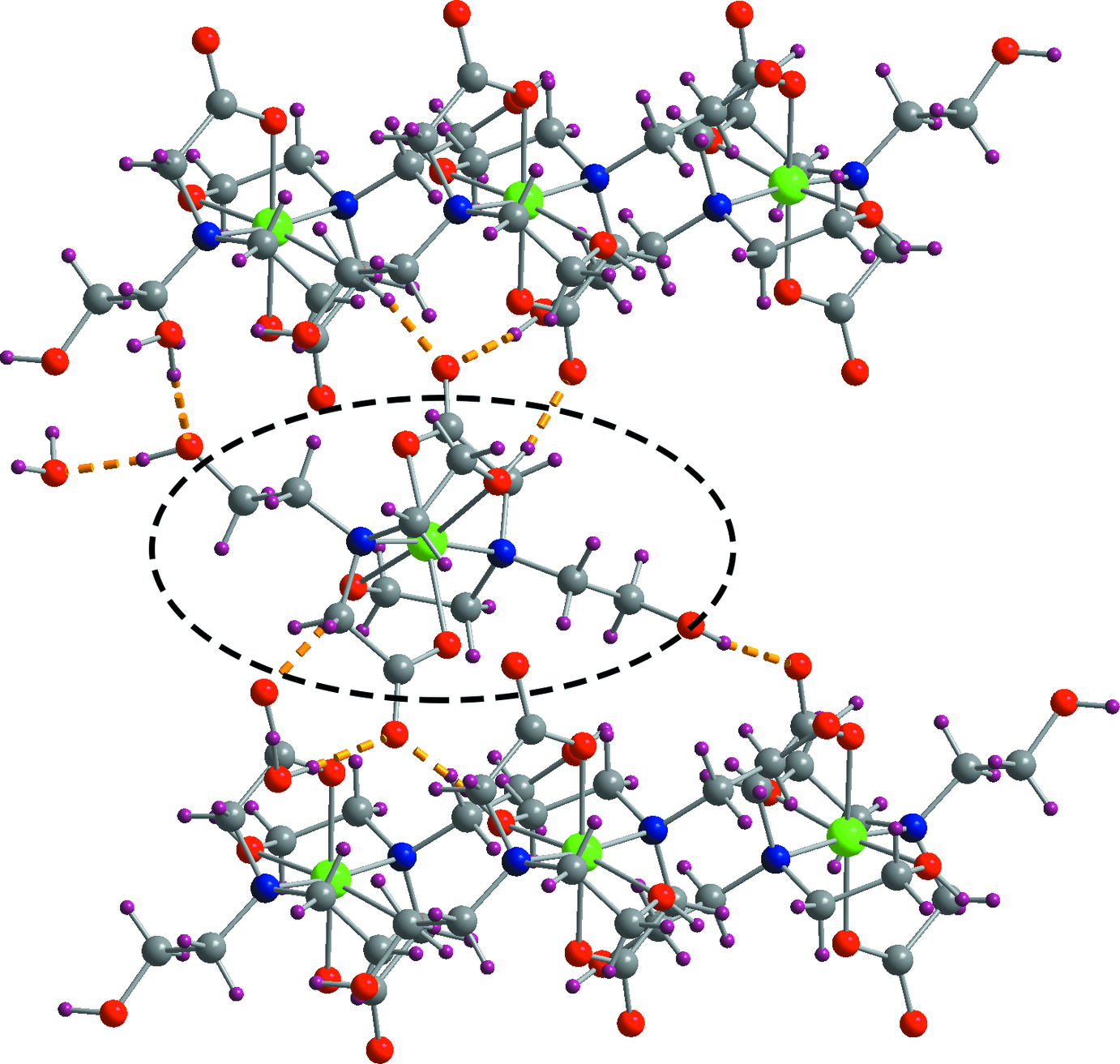

Supplement: Supplementary file 6 [file e-71-0m199-fig3.tif]
